# Supplementary material for: Ultrasensitive detection of miRNA with an antimonene-based surface plasmon resonance sensor
Source: Nat Commun. 2019 Jan 3;10:28. doi: 10.1038/s41467-018-07947-8 (PMC6318270; doi:10.1038/s41467-018-07947-8)
Supplement: Supplementary file 1 — Supplementary Information [file 41467_2018_7947_MOESM1_ESM.pdf]

Supplementary Information

Ultrasensitive detection of miRNA with an antimonene-based surface plasmon resonance sensor

Tianyu Xue,[1] Weiyuan Liang,[1] Yawen Li,[2] Yuanhui Sun,[2] Yuanjiang Xiang,[1] Yupeng Zhang,[1] Zhigao Dai,[3,4] Yanhong Duo,[1] Leiming Wu,[1] Kun Qi,[1] Bannur Nanjunda Shivananju,[1] Lijun Zhang,[2]* Xiaoqiang Cui,[2]* Han Zhang,[1]* Qiaoliang Bao[1,3]*

1. Key Laboratory of Optoelectronic Devices and Systems of Ministry of Education and Guangdong Province, College of Electronic Science and Technology and College of Optoelectronics Engineering, Shenzhen University, Shenzhen 518060, P. R. China

2. School of Materials Science and Engineering and Key Laboratory of Automobile Materials of MOE, Jilin University, Changchun, Jilin 130012, P. R. China

3. Department of Materials Science and Engineering, ARC Centre of Excellence in Future Low-Energy Electronics Technologies (FLEET) Monash University, Clayton, Victoria 3800, Australia

4. School of Printing and Packaging and School of Physics and Technology, Wuhan University, 299 Bayi Road, Wuchang District, Wuhan 430072, Hubei Province, P. R. China

Correspondence and requests for materials should be addressed to L. Z. (lijun_zhang@jlu.edu.cn), X. C. (e-mail: xqcui@jlu.edu.cn), H. Z. (e-mail: hzhang@szu.edu.cn) or Q. B. (e-mail: qiaoliang.bao@monash.edu).

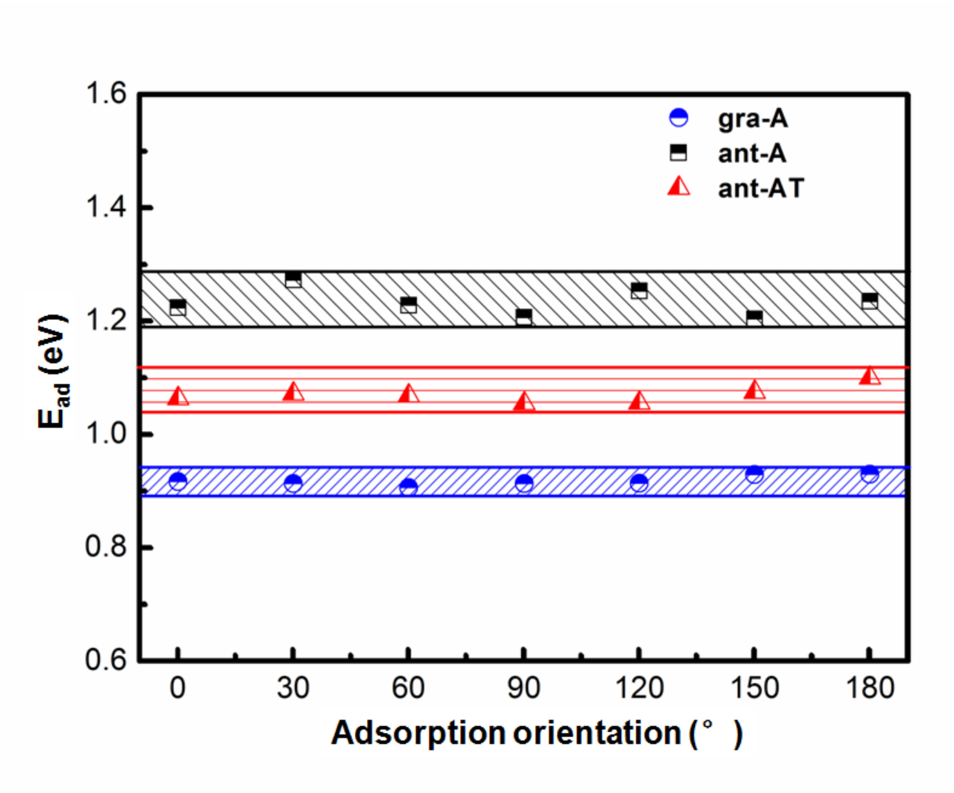

Supplementary figure 1. The DFT calculation for the nucleobases on top of antimonene/graphene with varied adsorption orientations. We calculated the single-stranded DNA (A nucleobases) on graphene and antimonene respectively, and the double-stranded DNA (A-T base-pairs) on antimonene with the different orientation angles changing from 0°, 30°, 60°, 90°, 120°, 150°, to 180°. The calculated adsorption energies of the three cases are shown. Turing to the work function results, our calculations indicate the maximum 2% change with different orientations for all the cases.

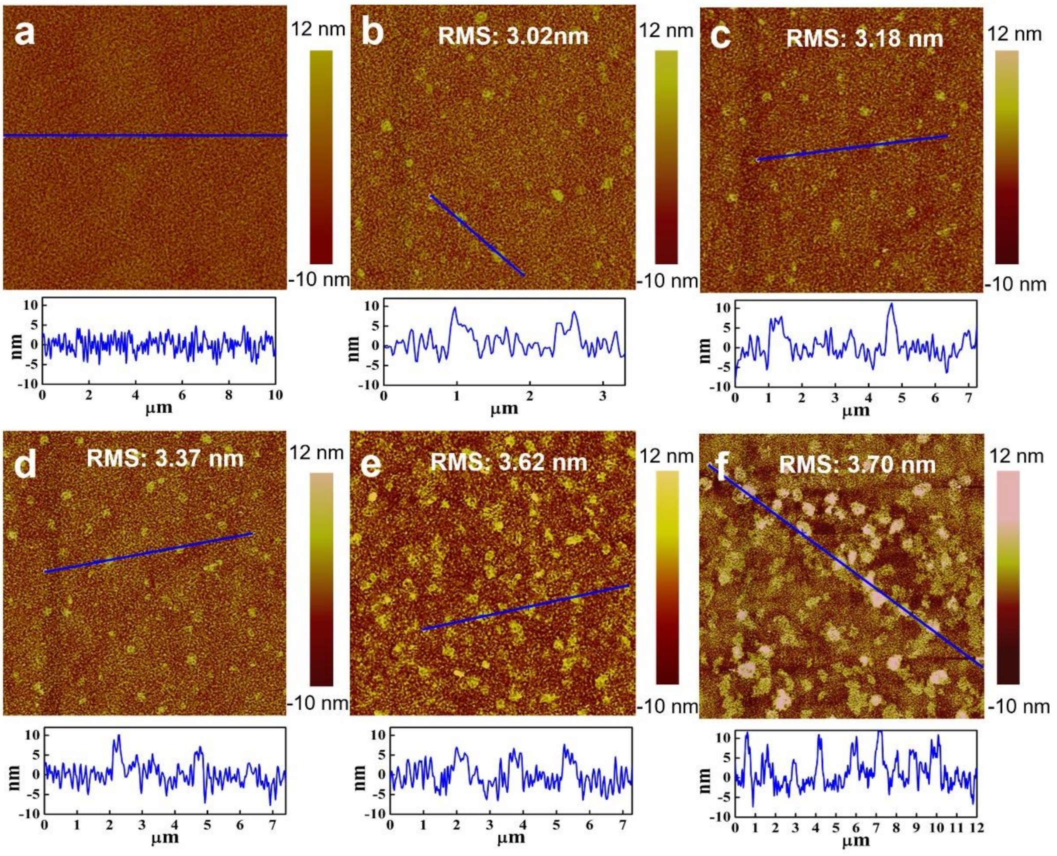

Supplementary figure 2. AFM images and height profile of antimonene film deposited using the electrostatic layer-by-layer method. a-f, Antimonene thin films with varied numbers of layers from 0 layer to 5 layers.

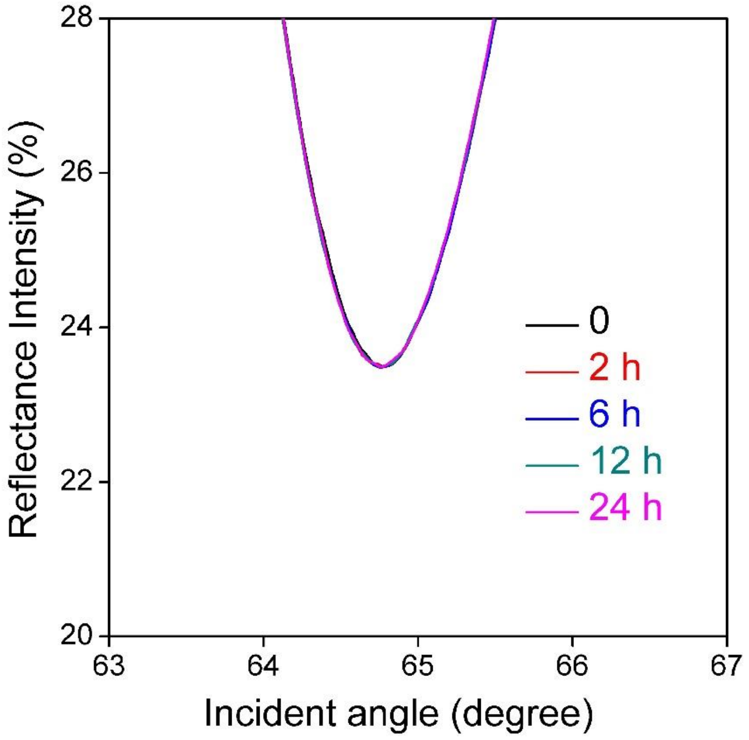

Supplementary figure 3. The SPR spectra for long time chemical durability of antimonene nanosheets. The SPR curves of antimonene-probe AuNR-ssDNA at the water solution for 2 h, 6h, 12h, and 24h.

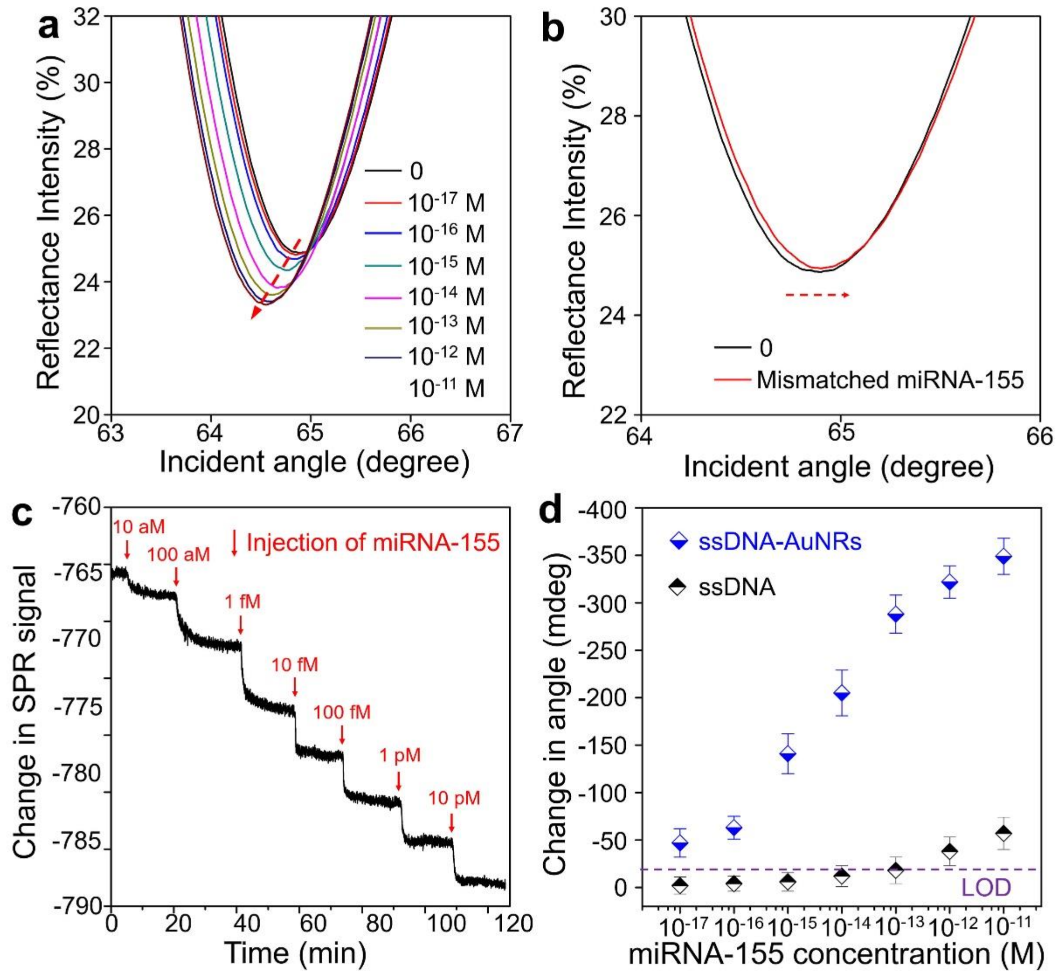

Supplementary figure 4. Sensing of miRNA-155 using an antimonene SPR sensor. **a,** SPR spectra with miRNA concentrations ranging from 10^{-17} to 10^{-11} M obtained using AuNR amplification. The arrow denotes the shift in the SPR angle. **b,** SPR curve change of miRNA-155 containing one mismatched nucleobase. **c,** Real-time SPR response of ssDNA-AuNRs desorption from the antimonene surface. **d,** The relationship between the SPR angle and miRNA-155 concentration. Each point corresponds to an SPR angle shift for the indicated concentrations of miRNA-155. All error bars is the standard error of SPR angle shift from five data points.

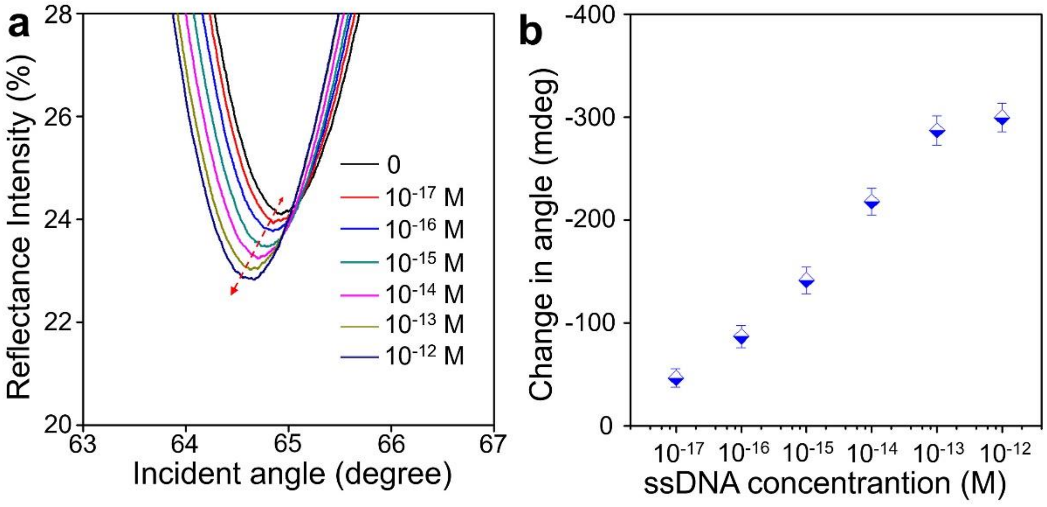

Supplementary figure 5. Sensing of ssDNA using an antimonene SPR sensor. a, SPR spectra with ssDNA concentrations ranging from 10^{-17} to 10^{-12} M obtained using AuNR amplification. The arrow denotes the shift in the SPR angle. **b,** The relationship between the SPR angle and ssDNA concentration. Each point corresponds to an SPR angle shift for the indicated concentrations of ssDNA. All error bars is the standard error of SPR angle shift from five data points.

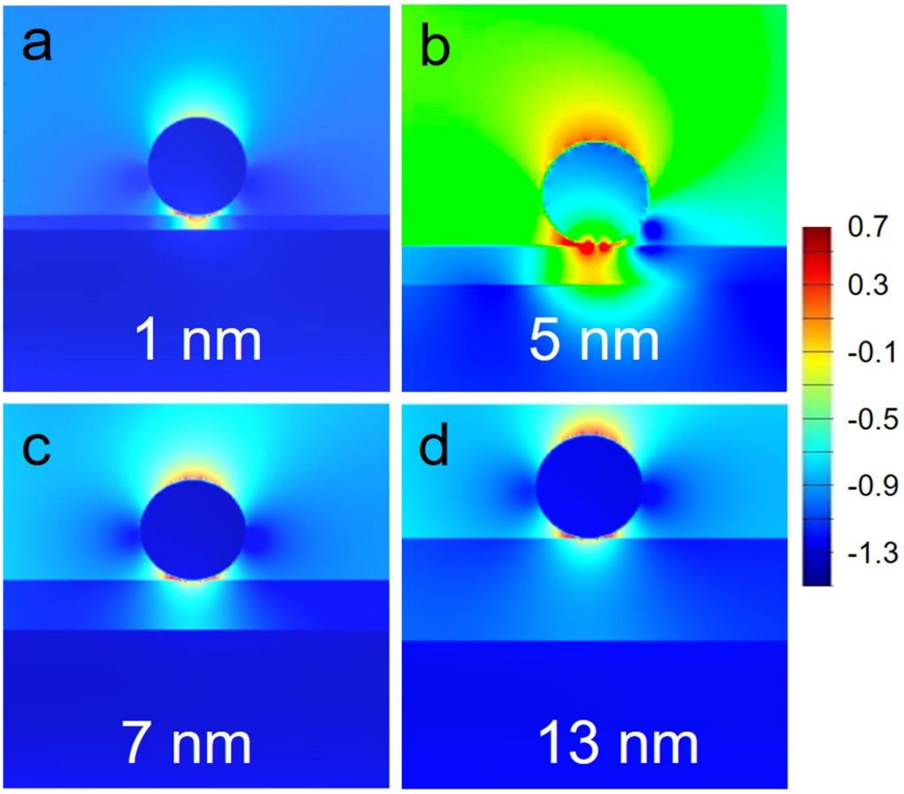

Supplementary figure 6. The local electric field distribution around AuNRs placed on antimonene nanosheets with different thicknesses. a, A antimonene film with the thickness of 1 nm is placed between the gold film and AuNRs. **b,** A antimonene film with the thickness of 5 nm is placed between the gold film and AuNRs. **c,** A antimonene film with the thickness of 7 nm is placed between the gold film and AuNRs. **d,** A antimonene film with the thickness of 13 nm is placed between the gold film and AuNRs. Based on above simulation, it can be seen that the local electric field is the strongest when the antimonene thickness is 5 nm.

Supplementary table 1. Oligonucleotide sequences used in the experiments.

Name	Sequence (5'→3')	Length
miRNA-21	UAG CUU AUC AGA CUG AUG UUG A	22
mismatched miRNA-21	UAG CUU AUC AGG CUG AUG UUG A	22
csDNA-21	SH - TCA ACA TCA GTC TGA TAA GCT A	22
miRNA-155	UUA AUG CUA AUC GUG AUA GGG GU	23
mismatched miRNA-155	UUA AUG CUA AUG GUG AUA GGG GU	23
csDNA-155	SH - ACC CCU AUC ACG AUU AGC AUU AA	23
ssDNA	GCT AGA GAT TTT CCA CAC TGA CT	23
csDNA	AGT CAG TGT GGA AAA TCT CTA GC	23
